# Oral–visceral iatrogenic Kaposi sarcoma following treatment for acute lymphoblastic leukemia: a case report and review of the literature

**DOI:** 10.1186/s13256-022-03620-3

**Published:** 2022-11-04

**Authors:** Richard Nyeko, Fadhil Geriga, Racheal Angom, Joyce Balagadde Kambugu

**Affiliations:** 1 Department of Paediatric Oncology, Uganda Cancer Institute, P.O. Box 3935, Kampala, Uganda; 2 Department of Paediatrics and Child Health, Lira University, P.O. Box 1035, Lira, Uganda

**Keywords:** Kaposi sarcoma, Acute lymphoblastic leukemia, Secondary malignancy, HHV-8, Pediatric case report

## Abstract

**Background:**

There have hardly been any reported cases of children presenting with Kaposi sarcoma as a second malignancy following treatment for acute lymphoblastic leukemia outside a transplant setting.

**Case presentation:**

We report a case of a 5-year-old boy of Bantu origin, which, to our knowledge, could be only the second reported case of oral–visceral Kaposi sarcoma after acute lymphoblastic leukemia treatment. The patient presented with a 1-month history of progressive, non-painful, soft tissue oral mass, 1 month after completing treatment for high-risk acute lymphoblastic leukemia. He was successfully treated for Kaposi sarcoma on a two-drug regimen (bleomycin and vincristine) with good clinical response.

**Conclusion:**

Visceral Kaposi sarcoma as a second malignancy may occur after pediatric acute lymphoblastic leukemia treatment, but its rarity makes it unlikely to raise suspicion among clinicians, thus precluding early diagnosis and treatment. We recommend routine evaluation for Kaposi sarcoma lesions in children undergoing long-term surveillance following treatment for childhood acute leukemia.

**Supplementary Information:**

The online version contains supplementary material available at https://doi.org/10.1186/s13256-022-03620-3.

## Introduction

Kaposi sarcoma (KS), as a second malignancy following treatment for acute lymphoblastic leukemia (ALL), particularly in the absence of transplant, has rarely been reported in children [1], and depreciates ALL treatment outcomes, especially in low-resource settings where survival generally remains low. First described by Moritz Kaposi in 1972 [2, 3], KS is an angioproliferative multicentric disease of lymphatic endothelium-derived cells. It is classified into four distinct clinicopathological subtypes based on the clinical condition in which it develops: (1) the classic or sporadic form mostly occurs in lower extremities in elderly patients of Mediterranean or Jewish origin, (2) the endemic form which is prevalent in middle-aged adults and children from sub-Saharan Africa, (3) the human immunodeficiency virus/acquired immunodeficiency syndrome (HIV/AIDS)-associated or epidemic KS, and (4) the iatrogenic form of KS, which is associated with immunosuppressive therapies for transplants or malignancies and other chronic inflammatory diseases [4, 5]. These variants portray different courses and epidemiology, but with similar histological features, and human herpes virus-8 (HHV8) is considered the causative agent for all forms of KS [4, 5].

The iatrogenic variant of KS occurs in patients who are immune-suppressed following organ transplant (particularly kidney transplants), chemotherapy, or rheumatologic disease [6], usually appearing a year after the first administration of the drugs. However, the occurrence of KS in children as a second primary malignancy following acute lymphoblastic leukemia (ALL) therapy in general, and outside transplant settings in particular, are scarce. Herein we report a case of iatrogenic KS in a 5-year-old boy, who presented with oral KS lesions (with visceral involvement) following ALL treatment (2 years and 8 months from the time he was first diagnosed with and initiated treatment for ALL).

## Case report

**Fig. 1:**
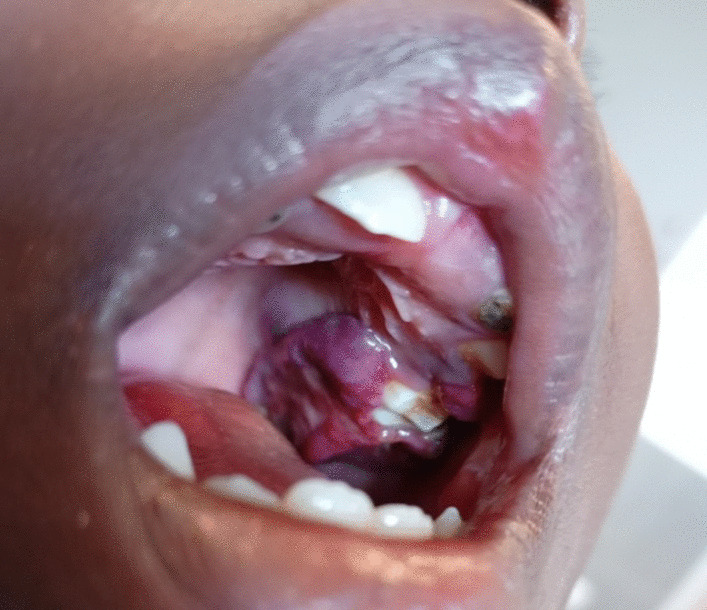
Iatrogenic oral KS lesions in a 5-year-old patient

A 5-year-old boy, of Bantu ethnicity, presented with a 1-month history of progressive, non-painful, soft tissue mass in the left palatal ridge, first noted in July 2021. He was otherwise well and had no reported constitutional symptoms, no jaw swelling and no abdominal or central nervous system symptoms. He had been earlier diagnosed with pre-B ALL [high risk owing to a high white blood cell count, according to the National Cancer Institute (NCI) criteria] at 3 years of age, for which he was treated at our center according to local protocol (Additional file 1) between November 2018 and June 2021, with well-achieved remission [minimal residual disease (MRD) negative], and had started long term surveillance. He did not receive irradiation or hematopoietic stem-cell transplantation during the first-line ALL treatment. His family and psychosocial history were unremarkable, and he had largely enjoyed normal growth, development, and health. A physical examination revealed a bluish–purplish mass in the left palatal ridge (Fig. 1) and no other significant findings (general or systemic).

**Fig. 2:**
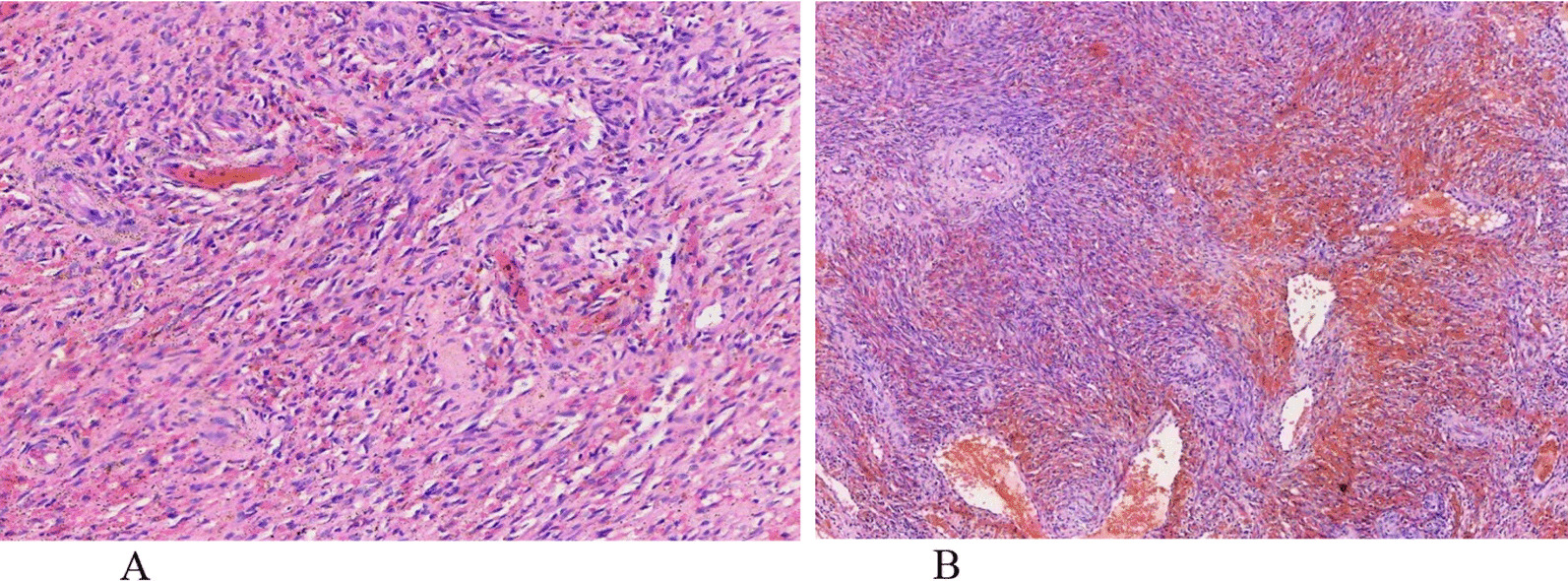
Histopathology (H & E staining) [40 ×]. **A** A tumor composed of nodules and spindle-shaped cells with mild atypia, **B** extravasated blood cells and proliferating vascular slits

A biopsy of the suspicious lesion was taken, and the histology was compatible with KS nodular stage. The hematoxylin and eosin (H & E) stained sections, illustrated in Fig. 2, showed a tumor composed of nodules of spindle-shaped cells with mild atypia (Fig. 2A) and extravasated blood cells and proliferating vascular slits (Fig. 2B).

**Fig. 3:**
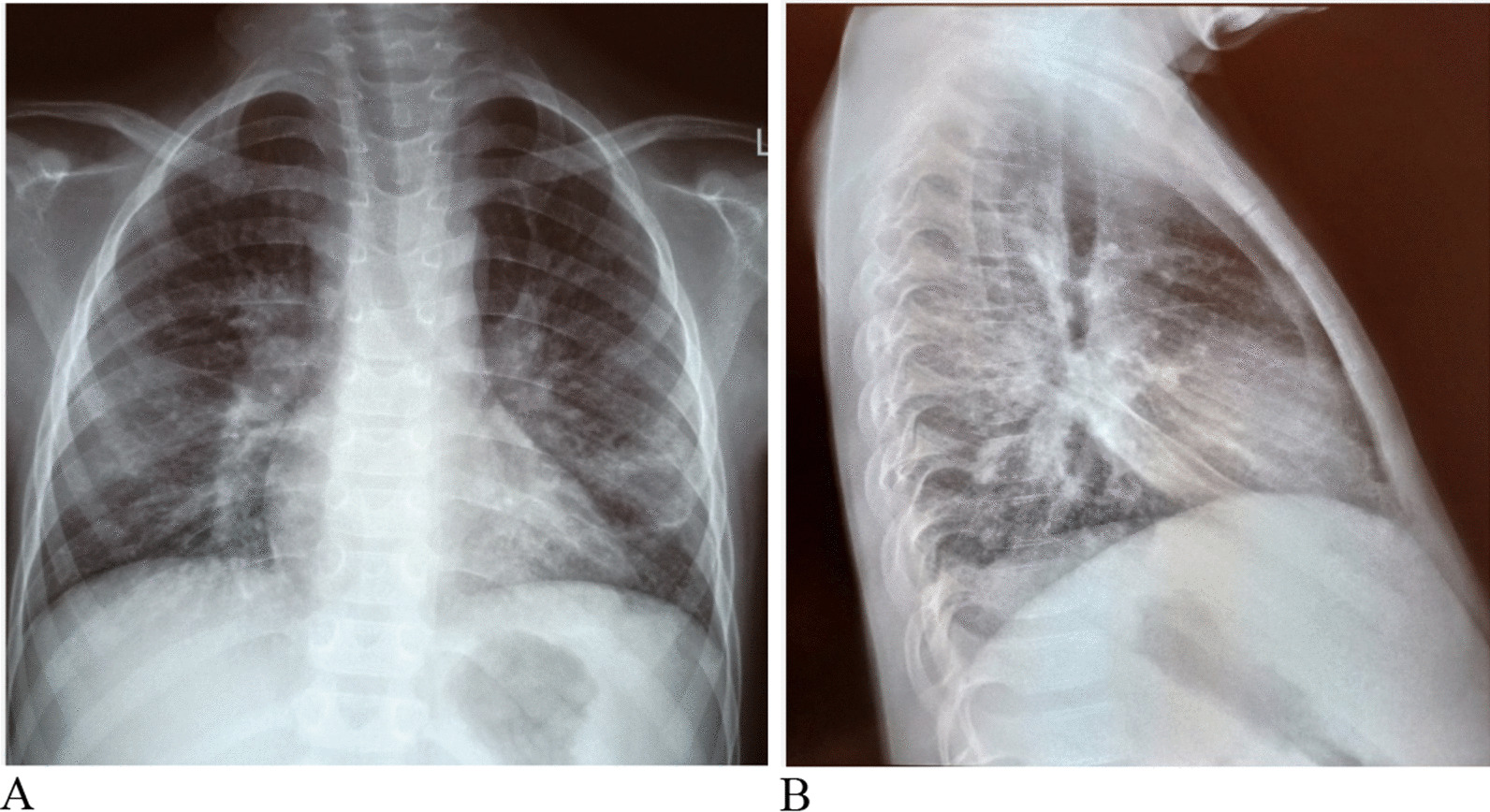
Chest radiograph. **A** Antero-posterior view, **B** lateral view, showing normal mediastinal and hilar regions.

An abdominal ultrasound scan revealed multiple enlarged intra-abdominal lymphadenopathies (para-aortic and mesenteric), the largest approximately 2.5 cm in longest diameter. Occult fecal blood was positive. The plain chest radiograph was largely normal (Fig. 3).

**Fig. 4:**
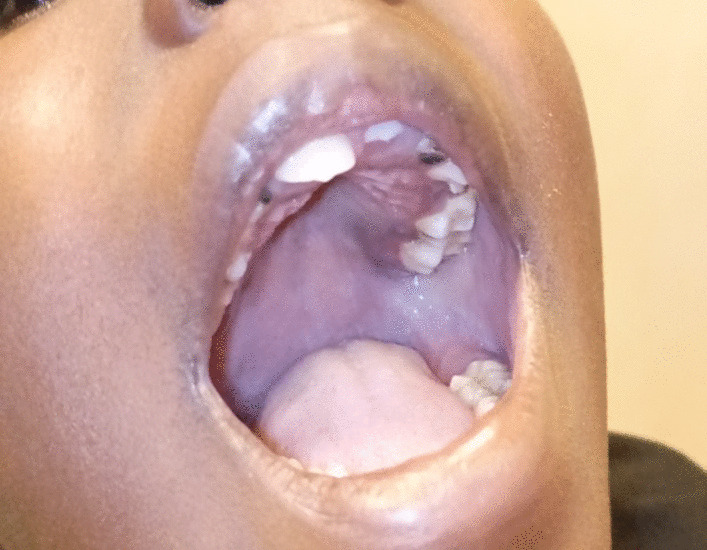
Complete resolution of oral KS after chemotherapy

The complete blood count, coagulation profile, and general chemistry were normal. The serological test for human immunodeficiency virus (HIV) antibody was negative. HHV8 assay and other immunological tests could not be performed at our center because of the lack of a testing facility. The final pathology diagnosis was iatrogenic Kaposi sarcoma. The patient was started on bleomycin (B) 10 IU/m^2^ 3 weekly and vincristine (V) 1.5 mg/m^2^ weekly (BV) according to local protocol, with complete clinical remission after four cycles of chemotherapy (Fig. 4)—defined as the disappearance of all clinical manifestations, including tumor-associated edema. The child’s clinical course was monitored by routine physical assessment and basic imaging studies.

The repeat abdominal ultrasonography and chest radiograph were normal, and the fecal occult blood test was negative by the fourth cycle of chemotherapy. He went on to receive a total of six cycles of the BV treatment regimen and continues to be monitored with regular evaluation.

## Discussion

This case is unique in the following aspects: While several cases of transplant-related iatrogenic KS have been described [7–9], reports on the occurrence of KS in children as a second primary malignancy following acute lymphoblastic leukemia therapy outside transplant settings are scarce. This is possibly only the second reported case of oral/visceral KS as a second malignancy in a child following ALL treatment, occurring outside the transplant setting. Further, KS has been infrequently reported as a complication following therapy of ALL in children.

Kaposi sarcoma (KS), first described by Moritz Kaposi in 1872 [2, 3], is a spindle-cell, slowly progressive multifocal lymphoangioproliferative neoplasm induced by a gamma herpes virus, the human herpes virus-8 (HHV-8), in predisposed individuals [4, 10]. There are four known clinical variants of KS, with different courses and epidemiology, but similar etiology: (a) epidemic or AIDS-associated KS, (b) classic KS (in elderly Mediterranean men), (c) endemic or African KS, and (d) iatrogenic KS, affecting immunosuppressed patients and organ transplant recipients [4, 10].

Infection with HHV-8 underlies a common etiology of all the subtypes of KS. However, though considered necessary, HHV-8 is not sufficient for the development of sarcoma, implying other factors (genetic, immunological, and environmental) are also necessary for the pathogenesis, together with immunosuppression [4, 10–13]. The iatrogenic form of KS, due to immunosuppression, is typically associated with the use of immunosuppressive therapy in organ transplant recipients (particularly kidney transplants). It can also occur in patients receiving immunosuppressive treatments for proliferative disorders/malignant processes or immune-mediated diseases [4, 6, 14, 15] as well as the use of oral corticosteroids and other immunosuppressive agents [6, 16, 17], with KS appearing usually about a year after the first administration of the drugs in these cases.

Here we report a case, which according to our knowledge from the review of literature, could be only the second reported case of Kaposi sarcoma following treatment for ALL, occurring outside the setting of a transplant, and the first with iatrogenic KS developing just over 2 years of ALL diagnosis in a child. One earlier documented case in the literature was of a 5-year-old girl who developed visceral KS 7 years after ALL treatment and clinical remission, and was only identified in autopsy findings [1]. A latter report by Sala *et al.* [18] was of a 10-year-old who developed KS just over a year following partially matched allogeneic hematopoietic stem cell transplant (HSCT) for relapsed acute myeloid leukemia.

In a subsequent review of 61 reported cases of second neoplasms in children and adults with ALL, the most frequent second tumors were other acute leukemias [mainly acute myeloid leukemia (AML)], lymphomas [mainly Hodgkin disease (HD)], and other solid tumors [19] other than KS. The majority of the secondary KS reported to date were in adults, mostly following solid organ transplants and immunosuppressive therapy for chronic inflammatory diseases such as systemic lupus erythematosus [5], Crohn’s disease [20], asthma [21], Wegener’s granulomatosis [22], or following transplant [23]. For instance, in Iran, Ramzi and colleagues reported a case of iatrogenic KS in a 44-year-old female after a hematopoietic stem cell transplant (HSCT) for acute myelogenous leukemia (AML) relapse [24].

The pathogenic mechanism underlying KS in patients treated for ALL is still not well known, though decreased immunity seems to be the overarching factor. By and large, the current most accepted hypothesis for the development of iatrogenic KS, in general, is supposed to stem from a weakening of the immunological surveillance system by immunosuppressive agents, resulting in the reactivation of a pre-existent HHV-8 infection and giving rise to the proliferative transformation of the infected endothelial cells [14]. In this respect, the pathogenesis is believed to relate to inhibition of transforming growth factor (TGF)-β and reactivation of latent HHV-8 infection, which leads to the induction of angiogenesis [4]. As postulated for chronic lymphocytic leukemia (CLL), the role of an ineffective T-cell response to HHV-8 latent infection, consequent to the generally decreased immunity, cannot be ignored [10], but other risk factors probably exist. Additionally, carcinogenesis may be a feature of chemotherapy in cancer treatment [25] and, only in the case of some organ transplant recipients, HHV-8 seroconversion may follow the immunosuppression, suggesting infection from the donated organ [26].

In general, the clinical manifestation of iatrogenic KS tends to be exclusive to cutaneous and/or mucosal involvement, though widespread disseminated forms with visceral involvement, as was the case in our patient, may also occur [14]. The objective for the management of KS is to manage the symptoms as treatment is often not curative, and prognosis depends on the disease severity at presentation [27]. Patients with systemic disseminated KS, as in our case, require systemic chemotherapy [28]. The clinical manifestations of KS following significant immunosuppression generally resolve, in most cases, when the immunosuppressive therapy is changed, reduced, or discontinued [5]. However, the reduction or elimination of immunosuppression does not always lead to the resolution of KS [4], and maintaining immunosuppression frequently leads to further disease progression [14]. Literature and current data regarding the treatment of KS following ALL treatment in children are lacking. Sala *et al*. reported achievement of complete remission with pegylated lysosomal doxorubicin used to treat visceral KS in allogeneic haematopoietic stem cell transplant recipients, with a disease-free state after 33 months of ongoing follow-up [18]. Pegylated lysosomal doxorubicin was similarly effective in the treatment of AIDS-related Kaposi patients in a report by Di Lorenzo *et al*. [29]. However, as noted by Sala *et al.* in their report, the use of anthracylines in the treatment of KS following ALL treatment may be limited by the increased risk of cardiotoxicity from anthracyclines administered during the first-line treatment of acute leukemia [18].

Another agent, sirolimus, has shown mixed results when used in the treatment of dermal KS in post-renal transplant recipients. For instance, Stallone *et al.* [30], just like other authors [31, 32], demonstrated a potential role of sirolimus in inhibiting the progression of KS in kidney transplant recipients, postulated to occur through the down-regulation of vascular endothelial growth factor (VEGF) and inhibition of tumor angiogenesis [30, 33]. On the contrary, Tanja Graf *et al*. reported no response to sirolimus when used in the treatment of cutaneous KS in a 36-year-old renal transplant recipient [34]. Here we report a case of successful use of a combination of bleomycin and vincristine in the treatment of oral–visceral KS in a child following ALL treatment, with remarkable improvement noted by the fourth of the 3-weekly cycles of this regimen, with disease-free status after about 1 year of ongoing follow-up. We believe this presents a significant result, especially for the treatment of KS in a resource-limited setting, because of the main advantages of this regimen, which are low cost, ease of administration, and minimal adverse effects.

This case report derives its strength from its uniqueness—being one of very few reported cases of oral/visceral KS as a second malignancy in a child following ALL treatment that occurs outside the transplant setting. The main limitations of this case report include the lack of an immunological study for HHV-8 and the short duration of patient follow-up, which limits the conclusion on long-term prognosis.

## Conclusion

Visceral KS as a second malignancy may occur after pediatric ALL treatment, but its rarity makes it unlikely to raise suspicion among clinicians, thus precluding early diagnosis and treatment. We recommend routine evaluation for KS lesions in children undergoing long-term surveillance following treatment for childhood malignancies, including acute leukemia. The administration of a combination of bleomycin and vincristine in the setting of discontinued immunosuppressive therapy may represent a relatively safe, effective therapeutic approach for these children, especially in low-resource contexts.

## Supplementary Information

**Additional file 1.** Acute lymphoblastic leukemia treatment protocol, Uganda Cancer Institute.

## Data Availability

Not applicable.

## Abbreviations

AIDS: Acquired immunodeficiency syndrome
ALL: Acute lymphoblastic leukemia
AML: Acute myeloid leukemia
HIV: Human immunodeficiency virus
H & E: Hematoxylin and eosin
HHV8: Human herpes virus 8
MRD: Minimal residual disease
KS: Kaposi sarcoma
