# Supplementary material for: Oral–visceral iatrogenic Kaposi sarcoma following treatment for acute lymphoblastic leukemia: a case report and review of the literature
Source: J Med Case Rep. 2022 Nov 4;16:405. doi: 10.1186/s13256-022-03620-3 (PMC9635084; doi:10.1186/s13256-022-03620-3)
Supplement: Supplementary file 1 — Additional file 1. Acute lymphoblastic leukemia treatment protocol, Uganda Cancer Institute. [file 13256_2022_3620_MOESM1_ESM.pdf]

Supplementary material 1

Acute lymphoblastic leukemia treatment protocol, Uganda Cancer Institute.

1. Induction

DRUG	ROUTE	DOSAGE			DAYS
Prednisone (Pred)	PO	60mgs/m^2/day (0 to< 10yrs) 40/m^2/day above 10 yrs. Max 100mg/d			1-28
Vincristine (VCR)	IV	1.5mgs/m^2/day (2mgs Max)			1, 8, 15, 22
Daunorubicin	IV	25mgs/m^2/day			1, 8, 15, 22
L-Asparaginase (Asp)	IM	6000IU/m^2/day			4-25 MWF x 6 doses
		**1-1.99yrs**	**2-2.99yrs**	**>3yrs**	
Arac	IT	30mgs	50mgs	70mgs	D_1
MTX	IT	8mgs	10mgs	12mgs	$D_{15, 29}$
Septrin		80mgs/m^2 (TMP)			D_{1-28}

2. Consolidation

DRUG	ROUTE	DOSAGE			DAYS
6-Mecarptopurine	PO	60mgs/m^2/Day			d_{1-28}
Cyclophosphamide	IV infusion	1000mgs/m^2/Day			d_1 d_{15}
Cytarabine	IV infusion	75mgs/m^2/Day			d_{1-4}, d_{8-11}, d_{15-18}, d_{22-25}
		**1-1.99yrs**	**2-2.99yrs**	**>3yrs**	
MTX	IT	8mgs	10mgs	12mgs	$D_{15, 29}$
Septrin		80mgs/m2 (TMP)			D_{1-28}

3. Interim maintenance 1 with capizzi

DRUG	ROUTE	DOSAGE		DAYS
VINCRISTINE (VRC)	IV	1.5mgs/m^2/day (2mgs max)		$d_{1, 11, 21, 31, 41}$
L-Asparaginase (Asp)	IM	6000IU/m^2/day		2-12 MWF x 6 doses
Methotrexate (MTX)	IV	100mgs/m^2		$d_{1, 11, 21, 31, 41}$
Methotrexate (MTX)	IT	**<u>Age</u>**<br>1-1.99<br>2-2.99<br>>3	**<u>Dosage</u>**<br>8mgs<br>10mgs<br>12mgs	d_{15}, Then d_{29}

4. Intensification (Re-induction)

DRUG	ROUTE	DOSAGE	DAYS		
Dexamethasone	PO	10mgs/m^2/Day	<12yrs<br>D_{1-21}<br>taper	>12yrs<br>D_{1-7} D_{15-21} D_{22-29}	
Vincristine	IV	1.5mg/m^2/Day	$D_{1, 8, 15}$		
Adriamycin	IV<br>Infusion	25mgs/m^2/Day	$D_{1, 8, 15}$		
L-Asparaginase	IM	6000IU/m^2/Day	D_{1-14} MWF x 6doses		
		**<u>1-1.99yrs</u>**	**<u>2-2.99yrs</u>**	**<u>>3yrs</u>**	
MTX	IT	8mgs	10mgs	12mgs	$D_{15,29}$
Septrin		80mgs/m^2<br>(TMP)	D_{1-28}		

5. Intensification (re-consolidation)

DRUG	ROUTE	DOSAGE			DAYS
Cyclophosphamide	Infusion	1000mgs/m^2/Day			D_{29}
6-MP	PO	60mgs/ m^2/Day			D_{29-42}
Cytarabine	Infusion	75mgs/m^2/Day			D_{29-32}, D_{36-39},
L-asparaginase	IM	6000IU/m^2/Day			D_{43-53}, MWF x 6 doses
Vincristine	IV	1.5mgs/m^2/Day			D_{43}, D_{50}
		**<u>1-1.99yrs</u>**	**<u>2-2.99yrs</u>**	**<u>>3yrs</u>**	
MTX	IT	8mgs	10mgs	12mgs	$D_{43,57}$
Septrin		80mgs (TMP)			D_{1-28}

6. Interim maintenance 2 with capizzi

DRUG	ROUTE	DOSAGE	DAYS
VINCRISTINE (VRC)	IV	1.5mgs/m^2/day (2mgs max)	$d_{1, 11, 21, 31, 41}$
L-Asparaginase (Asp)	IM	6000IU/m^2/day	2-12 MWF x 6 doses
Methotrexate (MTX)	IV	100mgs/m^2	$d_{1, 11, 21, 31, 41}$
Methotrexate (MTX)	IT	**Age** / **Dosage**<br>1-1.99 / 8mgs<br>2-2.99 / 10mgs<br>>3 / 12mgs	d_{15}, Then d_{29}

7. Long term maintenance

DRUG	ROUTE	DOSAGE	DAYS
Dexamethasone (Dexa)	PO	6mgs/m^2/day	$D_{1-5, 29-33, 57-61}$
Vincristine (VCR)	IV	1.5mgs/m^2/day<br>(2mgs Max)	$D_{1, 29, 57}$
Mercaptopurine (6MP)	PO	75 mgs/m^2/dose	D_{1-84}
Methotrexate (MTX)	PO	20mgs/m^2/dose	Weekly D_{8-78} (exclude 29 for first 4 cycles)
Methotrexate (MTX)	IT	**Age** / **Dosage**<br>1-1.99 / 8mgs<br>2-2.99 / 10mgs<br>>3 / 12mgs	First 4 Cycles: D_{1}, Then D_{29}<br>Cycles >4: D_{1}
